# First-Principles Analysis of Vibrational Properties of Type II SiGe Alloy Clathrates

**DOI:** 10.3390/nano9050723

**Published:** 2019-05-10

**Authors:** Dong Xue, Charles W. Myles

**Affiliations:** Department of Physics and Astronomy, Texas Tech University, Lubbock, TX 79409-1051, USA; charley.myles@gmail.com

**Keywords:** alloy clathrate, mode Grüneisen parameter, negative thermal expansion, quasi-harmonic approximation

## Abstract

We have mostly performed vibrational studies of Type-II silicon-germanium clathrate alloys, namely, Si_136-x_Ge_x_ (0 < *x* ≤ 128), using periodic density functional theory (DFT). Our computed lattice constant for various stoichiometric amount, namely, *x*, of Ge agrees to some extent with the observed X-ray diffraction (XRD) data, along with monotonically increasing dependence on *x*. According to our bandgap energy calculation via Vienna *ab initio* simulation package (VASP), Si_128_Ge_8_ has a “nearly-direct” bandgap of approximately 1.27 eV, which agrees well with the previously calculated result (~1.23 eV), which was obtained using the Cambridge sequential simulation total energy package (CASTEP). Most of our first-principles calculations focus on exploring the low-energy transverse acoustic (TA) phonons that contribute dominantly to the induction of negative thermal expansion (NTE) behavior. Moreover, our work has predicted that the Si_104_Ge_32_ framework exhibits NTE in the temperature range of 3–80 K, compared to the temperature regime (10–140 K) of NTE observed in such pure Si_136_. It is posited that the increased number of Ge–Ge bonds may weaken the NTE effect substantially, as the composition, which is denoted as *x*, in Si_136-x_Ge_x_ is elevated from 32 (or 40) to 96 (or 104).

## 1. Introduction

In contrast to the diamond phase of silicon (*d*-Si), there are two forms of crystalline clathrate: Si_46_ (Type I) and Si_34_ (Type II). Each of these pure materials consists of a covalently bonded framework that is composed of polyhedron cage elements. The enlarged unit cell of the Type II clathrate framework contains 136 atoms, exhibits a face-centered cubic (FCC) lattice structure and contains 20- and 28-atom cages that are connected periodically in a 4:2 ratio [1]. Growing interest in this expanded-volume silicon has arisen for two main reasons: the confirmed existence of superconductivity in metal-doped clathrate, namely, Ba_x_Na_y_Si_46_ [2,3,4,5], and the massive studies that have been conducted on efficient thermoelectric (TE) performance with guest-filled Si clathrates, which display glass-like thermal conductivity while behaving as a crystalline-cubic material [6,7,8]. Specifically, the efficiency of a TE device is manifested by the material’s figure-of-merit, *ZT ≡ σS^2^T/κ*, where *σ* denotes the electrical conductivity, *S* is the Seebeck coefficient; *T* is the absolute temperature, and *κ* is the thermal conductivity. An effective way of enhancing *ZT* is through reducing the phonon thermal conductivity by nanostructuring [9], alloying [10], or introducing cage-like configuration that encapsulates rattling atoms, such as Si- or Ge-based clathrate compounds [11].

At present, many reports have discussed the electronic and thermodynamic properties of Si- and Ge-based Type II clathrate compounds [12,13,14,15,16,17] with the objectives of identifying prominent TE materials and gaining insight into interesting properties such as anomalous thermal expansion. One characteristic of the ideal TE candidate is minimal “glass-like” lattice thermal conductivity, which can originate from the scattering of acoustic phonons by the guest atoms [18,19] while satisfying the “Phonon Glass Electron Crystal” (PGEC) paradigm, which was proposed by G. A. Slack [20,21]. Motivated by this concept, work on the Si-based Type II materials, namely, Cs_8_Ga_8_Si_128_ (Rb_8_Ga_8_Si_128_), and Ge-based Type I materials, namely, Ba_8_Ga_16_Ge_30_ (Ba_8_Ga_16_Si_5_Ge_25_), has been presented by K. Biswas et al., in which the existence of the very low frequency rattling modes of guests, which contribute to the suppression of the phonon thermal conductivity, are demonstrated [22,23]. In addition, using density functional theory (DFT) within the local density approximation (LDA) approach, Tang et al. examined the thermal properties of the pure Si_136_ framework (which is sometimes denoted as Si_34_) and reported the negative thermal expansion (NTE) phenomenon in the temperature range of 10–140 K in experimental as well as theoretical manner [24]. More information about measured thermal properties of Si_136_ and bulk modulus with respect to Na_x_Si_136_ (0 < *x* < 24) (and low-Na Si clathrate-II compositions) are referred to articles [25,26].

Recently, alloy clathrates have attracted wide research attention, and are a class of materials that possess a crystalline framework that is composed of more than one Group IV element (e.g., Si, Ge and Sn). In this paper, the study of alloyed clathrates that are based on a mixture of Si and Ge is of great importance. Our calculational work is centered on these semiconducting clathrates, which have technological potential in both optical and electrical applications [27,28,29]. The SiGe binary clathrate compounds that contain metallic fillers in the endohedral sites of the polyhedron cage have been experimentally studied by Herrmann et al. [30]. In addition, Moriguchi et al. has investigated the energetics and electronic properties of the Si_136-x_Ge_x_ (0 ≤ *x* ≤ 136) system via first-principle codes [31]. Their work predicts direct and “nearly-direct” wide band gap whose value ranges from 1.2 to 1.3 eV according to the Ge content (*x*) which is in the range of 0 and 40. We adopted their atomic compositions of Si_136-x_Ge_x_ to consider a full symmetry structure with equivalent Wyckoff sites, thereby facilitating the smooth initiation of our DFT calculations. Moreover, the physical origin of the low-temperature negative thermal expansion in Si_136-x_Ge_x_ (0 ≤ *x* ≤ 128) remains ambiguous and is under discussion [32,33,34]. To the best of our knowledge, one primitive model that has been applied to theoretically survey anharmonic thermodynamics that correspond to NTE behavior exploits the volume-dependent mode Grüneisen parameter (*γ*_i_), which quantifies the deviation of the collective vibration of the lattice structure from simple harmonic oscillations. The measured shift of Raman lines under varied pressure also provides an experimental approach for examining the Grüneisen parameters when they are related to low-lying optical and acoustic modes of Na_1_Si_136_ [35].

In this work, we present an *ab initio* computational study on vibrational and thermal properties and their intrinsic relations to the finite-*T* NTE phenomenon based on the optimized geometry of Si_136-x_Ge_x_ with respect to various stoichiometric compositions. The quasi-harmonic approximation (QHA) method is the most effective tool, which considers only the volume dependence of phonon anharmonicity and assumes that the temperature has a negligible effect on the phonon vibrational frequencies in the case of thermal expansion. Our calculated negative mode Grüneisen parameters, which are calculated according to *γ*_i_ = −*ǝ*In*ω*_i_/*ǝ*In*V* and derived predominantly from heat-carrying transverse acoustic (TA) phonons that are located within the Brillouin zone (BZ), indirectly demonstrate the occurrence of NTE behavior. Here, vibrational mode frequency *ω*_i_ depends on volume *V* according to QHA. Furthermore, these first-principles results on *γ*_i_ remain to be comparable to the experimental Raman data [35,36]. The finite difference method (FDM) is utilized to estimate the above lattice dynamics quantities at *T* = 0, which explains how volume dilation results in varied dynamical matrix elements of phonons that are subjected to different bands. For instance, contraction of our supercell model yields larger mode frequencies of the longitudinal acoustic (LA) phonon and transverse optic (TO) phonon, which are located at the BZ boundary (X or L point). In addition, we report a theoretical analysis of the electronic properties of Si_136-x_Ge_x_ clathrates. Our calculated lattice constant values, along with the band gap as a function of the Ge concentration which was normalized, correlate well with data that were recently obtained using an X-ray diffraction (XRD) instrument [37].

## 2. Computational Approach

Our first-principles calculations are conducted using the Vienna *ab initio* simulation package (VASP) [38], which exploits the Ceperley-Alder exchange-correlation potential and pseudopotentials that are obtained via the projector augmented wave (PAW) method. The energy cutoff parameter that accounts for the plane-wave basis was selected as the default value (245.7 eV) when initiating the phonon calculations, which helps provide insight into the vibrational frequency of the Γ-point normal mode. A 4 × 4 × 4 Monkhorst-Pack *k*-point grid [39] is selected for Brillouin zone integration. 

The procedure of extracting electronic, vibrational and thermodynamic properties of the SiGe alloy clathrate from the periodic density functional theory computation is described as follows: The first step of geometry optimization is to relax the internal coordinates of the atoms, which are confined in a fixed unit cell of the materials. Then, the ground-state structural and electronic properties, such as the cohesive energy, were determined within the local density functional formalism. Next, a limited number of energy-volume (*E*, *V*) pairs were fitted to a 3rd-order Birch-Murnaghan equation of state (EOS) [40], thereby enabling the calculation of the global minimum energy and the equilibrium lattice parameter. In addition to optimizing the geometry of each of the studied alloy clathrates, electronic properties, including the Fermi energy level (*E*_F_), the electronic band structure (BS) and the electronic density of states (EDOS), are calculated in the framework of consistent structural settings.

To investigate the lattice dynamics of these Si-based clathrate compounds, a 2 × 2 × 2 Monkhorst-Pack *k*-point was applied to obtain Γ-point vibration frequencies and dispersive relations, which are derived from the harmonic force constant matrix. In addition, the thermodynamic properties that are related to phonon anharmonicity were evaluated with the aid of the QHA method: The fractional change in volume, namely, Δ*V*/*V,* which governs structural dilation or contraction, and the fractional change in the mode frequency are inspected to determine the microscopic Grüneisen parameter *γ*_i_. For this purpose, phonon calculations are repeated at three corresponding volume points that contain one equilibrium volume and two additional volumes that are slightly larger and smaller. Using the Feynman-Hellmann theorem, which is based on the FDM, the mode Grüneisen parameter of each phonon is evaluated by approximating the volume derivatives of dynamical matrix elements (*D_ij_*(***q***)) as Δ*D_ij_*(***q***)/Δ*V*.

## 3. Results and Discussion

### 3.1. Electronic Properties

**Figure 1:**
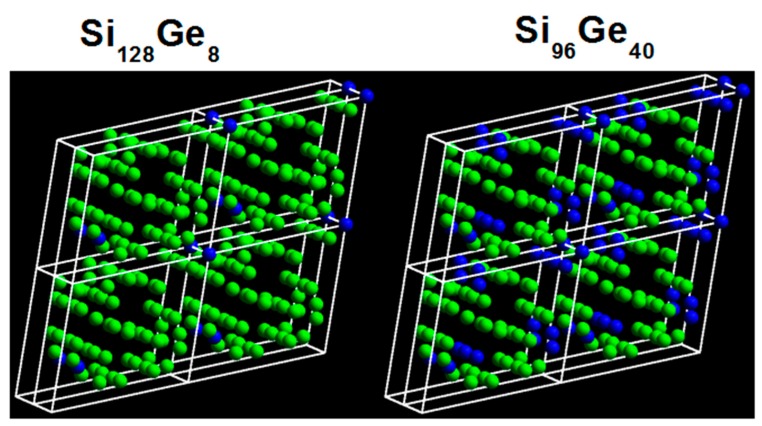
Cubic unit cells of Si_136-x_Ge_x_ (*x* = 8, 40) clathrate. The green solid balls represent the Si atoms while the blue solid ones denote the Ge atoms that act as substitutional framework hosts.

First, it is necessary to show the crystal structures with respect to Si_136-x_Ge_x_ (x = 8, 40) in Figure 1. Here, the specified cubic unit cells are schematically given for the configurations that consist of 256 and 192 silicon atoms out of 272 atoms per cell respectively. The blue solid balls in the figure denote the Ge atoms that replace the Si counterparts at all 8*a* Wyckoff sites in Si_128_Ge_8_ and at all 8*a* along with 32*e* Wyckoff sites in Si_96_Ge_40_. These clathrate alloys are expanded volume phase with sp^3^ tetrahedrally bonded framework.

Next, we performed the *ab initio* computation to determine various electronic properties of Type II SiGe alloy clathrates, which are structurally formulated in covalently bonding configurations and exhibit sp^3^-hybridized configurations. Previously, in synthesis work on Si_136-x_Ge_x_ (0 ≤ *x* ≤ 136) by Baranowski et al., their phase formats were classified into two categories according to the Ge composition, which is denoted as *x* [37]. Their study determined that the stoichiometric amount (*x*) of Ge for amorphous formation ranges from approximately 20.4 to 68. The amorphous region is likely caused by a miscibility gap. Analogous to those experimental results, the following figures present the results of our first-principles work on the composition-dependence of the lattice parameter and the bandgap for semiconducting [Si_1-x’_Ge_x’_]_136_ (0 < *x*’ < 1). Here, it is noticed that *x’* appearing in redefined chemical notation [Si_1-x’_Ge_x’_]_136_ remains equivalent to the ratio of Ge composition (*x*) to 136.

**Figure 2:**
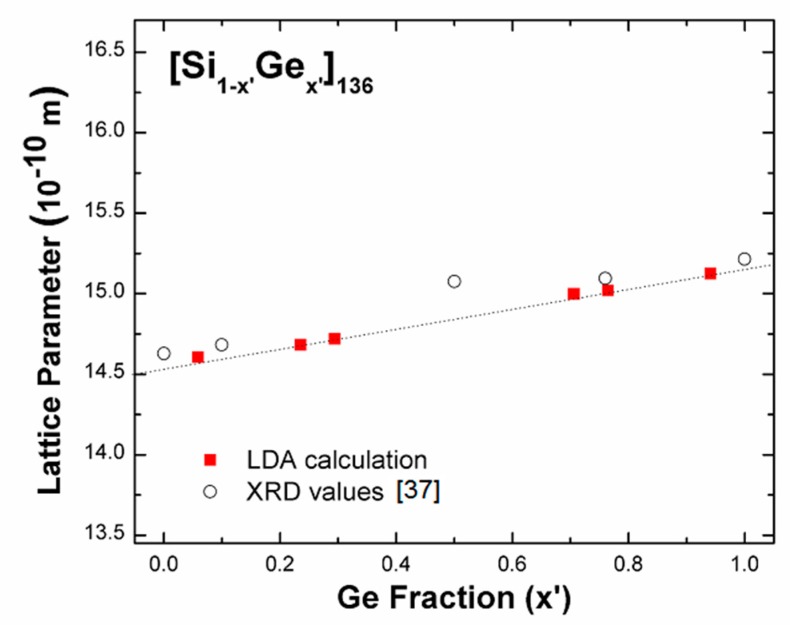
Local density approximation (LDA)-determined lattice parameter trend (red squares) as a function of the Ge mole fraction for [Si_1-x’_Ge_x’_]_136_, in comparison with XRD data (open circles). The dashed line that is drawn for the LDA data was obtained via a linear fitting procedure and acts as a guide for the eye. The unit 10^−10^ m is equal to 1 Å.

In Figure 2, the lattice parameter increases with the Ge content; a similar trend is observed between XRD data and our LDA work in the absence of an amorphous region (0.15 ≤ *x*’ ≤ 0.5). At various compositions of added Ge atoms (e.g., *x*’ = 0.15 and *x*’ = 0.5), the SiGe alloy clathrate exhibits a mostly crystalline phase with a small amount of amorphous background [37]. This demonstrates that the alloyed clathrate structures expand because of substitutional host atoms (Ge), in comparison with the pure Si_136_ framework. In addition, for *x*’ ~ 0.77, our equilibrium lattice constant is 15.05 Å, which is approximately 0.3% smaller than the XRD value [37]. In analogy to this, the previously calculated lattice constant of Si_136_ (14.56 Å) [1] is approximately 0.7% smaller than its experimental counterpart (14.63 Å) [41].

**Figure 3:**
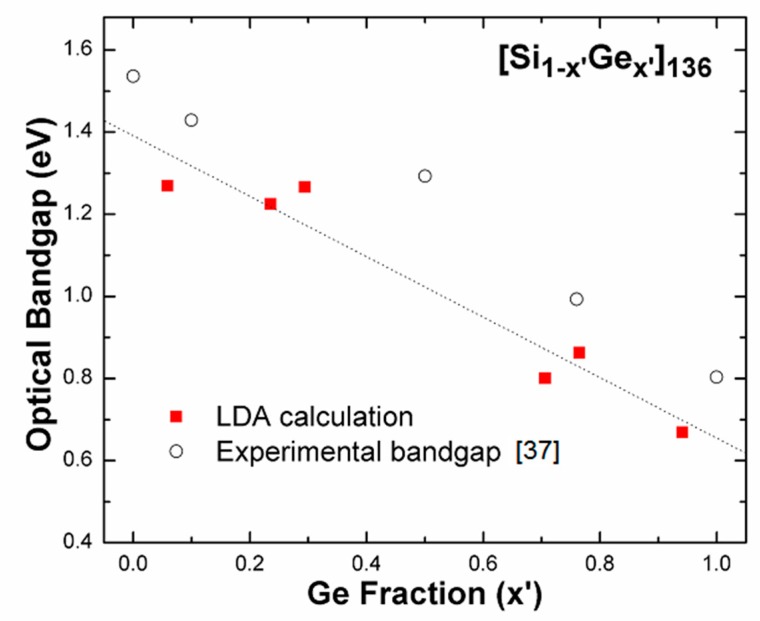
Comparison of the predicted band gap (red squares) and the experimentally measured results (open circles) for [Si_1-x’_Ge_x’_]_136_ (0 < *x*’ < 136). The dashed line acts as a guide for the eye.

**Figure 4:**
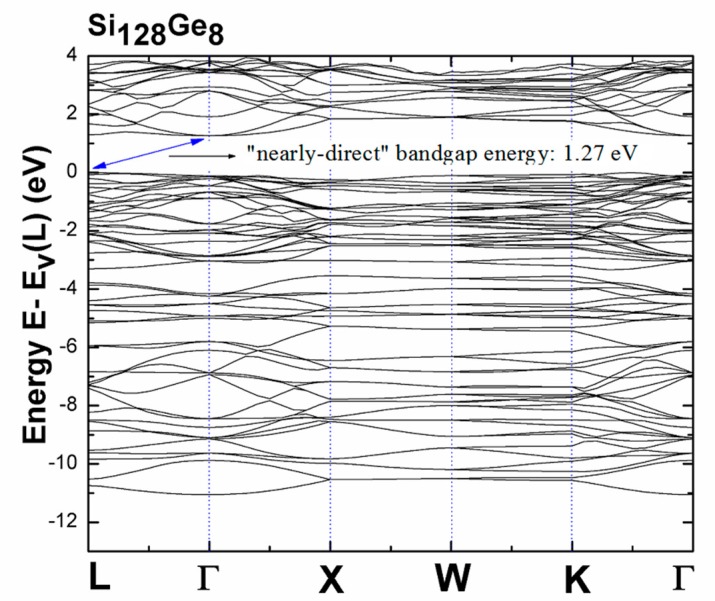
LDA-determined electronic band structures of Si_128_Ge_8_, where the zero of energy is set to be the valence band maximum at the L point (E_v_(L)). The “nearly-direct” bandgap is identified by blue arrow showing 1.27 eV.

A lower DFT-determined bandgap compared to the experiment result [37] in Figure 3 is expected no matter how *x’* appearing [Si_1-x’_Ge_x’_]_136_ changes, because the use of LDA formalism always causes the fundamental bandgap energy to be underestimated [42,43]. All optical band gap energies are measured from the top of the valence band at L, the zero of which remains stably fixed and independent of the Ge concentration. Additionally, we theoretically found that degeneracy of the lowest conduction band at L and Γ points is not noticeably distinguished in the presence of Si_128_Ge_8_ (see Figure 4), since eigenenergy of the conduction band edge at L is slightly higher (about only 30 meV larger) than eigenenergy of the conduction band edge at Γ point. Thus, we call this sort of bandgap a “nearly-direct” bandgap. Furthermore, the depicted band structure provided in Figure 4 shows that Si_136-x_Ge_x_ (*x* = 8) exhibits the “nearly-direct” behavior regarding band gap redefinition. The calculated magnitude of such band gap value turns out to be approximately 1.27 eV for Si_128_Ge_8_, which agrees well with the previous DFT result (~1.23 eV), which was obtained via the Cambridge sequential simulation total energy package (CASTEP) code [31].

**Figure 5:**
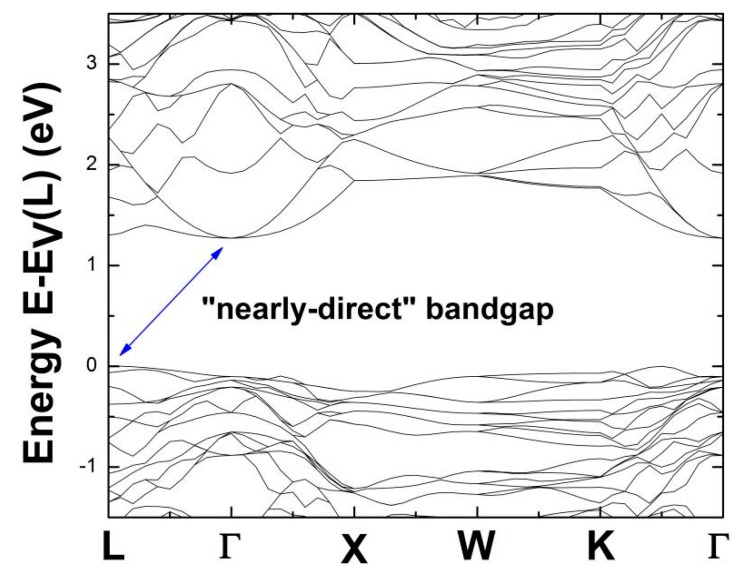
Detailed “nearly-direct” bandgap behavior within the energy (*E*-*E*_V_(L)) range between −1.5 eV and 3.5 eV.

In order to identify the detailed picture of “nearly-direct” band gap from the viewpoint of band structure (BS) given in Figure 4, we restrict the vertical scale about energy to range from −1.5 eV to 3.5 eV for the purpose of zooming into the BS in an intricate manner. Therefore, Figure 5 shows the apparent “nearly-direct” behavior of bandgap energy, because eigenenergy of conduction band edge at L is only about 30 meV larger than that of the conduction band edge at Γ point, compared to significantly large band gap value (about 1.27 eV).

### 3.2. Vibrational Properties

**Figure 6:**
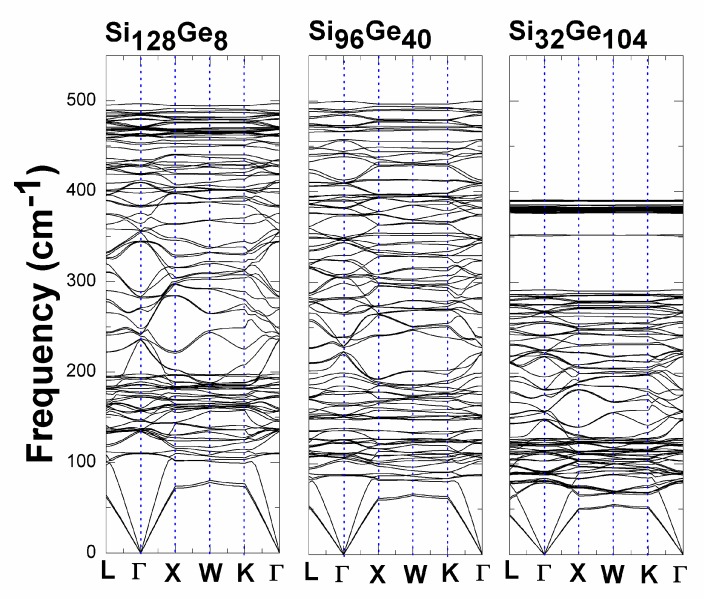
Dispersion relations of Si_136-x_Ge_x_ (*x* = 8, 40, 104) that were obtained via VASP.

The low-lying acoustic and optic mode regions are of greater importance than other portions of the predicted phonon-dispersion curves in Figure 6. Six phonon branches are primarily discussed here for each studied Si_136-x_Ge_x_ material (*x* = 8, 40, 104): the longitudinal acoustic, transverse acoustic (TA (1) & TA (2)) with double degeneracy along the specified direction, longitudinal optical (LO) and transverse optical (TO (1) & TO (2)) branches, which might coincide at various ***q***-points. To see the difference of the low-frequency portions (0–75 cm^−1^) of the dispersion relations for Si_128_Ge_8_ and Si_96_Ge_40_, we listed the frequency at L, X, W and K high-symmetry point in the following Table 1.

From the above Table, the vibrational frequency at fixed point decreases with the ascending order of Ge concentration *x*. Accordingly, the acoustic phonon speeds occur to be decreased with the increasing *x*. 

**Figure 7:**
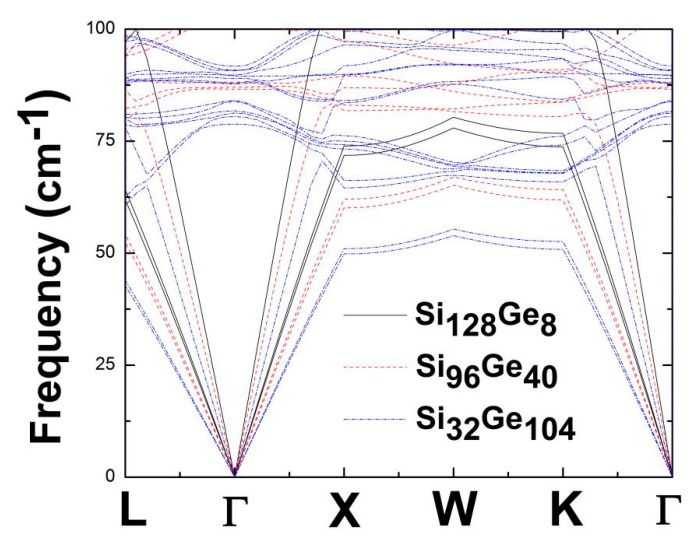
Low-frequency spectrum of Si_128_Ge_8_ (black solid line), Si_96_Ge_40_ (red dotted line), Si_32_Ge_104_ (blue dashed line).

Furthermore, the dispersion spectrum for Si_32_Ge_104,_ which is displayed in Figure 6, shows its compressed optical band region (71 cm^−1^~390 cm^−1^), for which the maximum frequency is reduced by approximately 21% compared to Si_128_Ge_8_ and Si_96_Ge_40_. Near the top of the optical bands, an extremely flat and dense phonon mode region is observed for Ge-dominant alloy Si_32_Ge_104_. This apparent reduction of the highest optical band in Si_32_Ge_104_ might be attributable to the raising number of loose Ge–Ge bond which force constant was previously reported to be around 10 eV/Å^2^ according to Dong’s work [44], compared to the “rigid” Si–Si bond, for which the effective force constant is approximately 24 eV/Å^2^ in Si_136_ [45]. Consequently, the existence of comparably weak coupling in the Ge–Ge bond might help suppress the sound speed of lattice phonons in Si_136-x_Ge_x_ when *x* abruptly jumps from 8 to 104. In addition to that, a much smaller frequency range is used in Figure 7 to illustrate how the low-lying acoustic phonon branches differ from each other among the alloyed clathrate system Si_136-x_Ge_x_ (*x* = 8, 40, 104). It is seen that each vibrational mode at specified point such as L, X, W, K possesses the frequency value appearing in the Table 1. Simultaneously, the acoustic phonon speed is also reduced accordingly as Si_128_Ge_8_ is switched to be Si_96_Ge_40_ to Si_32_Ge_104_.

We postulate that the collective motion of the framework atoms at each optimized geometry of Si_136-x_Ge_x_ is affected by the number of Ge–Ge bonds, from both vibrational and transport points of view. The models that were considered here for the composition of the Si_136-x_Ge_x_ system were suggested by Moriguchi et al., who stated that host atoms reside at three inequivalent sites (8*a*, 32*e*, and 96*g*) [31]. On the basis of this ideal *Fd3m* symmetry, they noted that the number of Ge–Ge bonds in each framework unit cell ranges from 0 in Si_128_Ge_8_ (and Si_104_Ge_32_) to 8 in Si_96_Ge_40_ and 36 in Si_40_Ge_96_ (and Si_32_Ge_104_); hence, they follow an ascending order. As many more and more Ge–Ge bonds begin to replace Si–Si bonds in Si_136-x_Ge_x_ framework with abruptly increasing stoichiometric amount of Ge, the existence of the relatively weakened bond-bond strength (lowered force constant) of Ge–Ge is anticipated to relate to the lowered absolute value of negative mode Grüneisen parameter found in transverse acoustic phonons. This leads to that the weighted average of *γ*_i_ switches its sign from negative to positive at low-temperature regime (e.g. 24–100 K) corresponding to the weakened NTE effect, when *x* is tuned from 8 (or 40) to 104. Detailed discussion on the derived mode Grüneisen parameter along with the macroscopic Grüneisen parameter is given in the following. 

**Figure 8:**
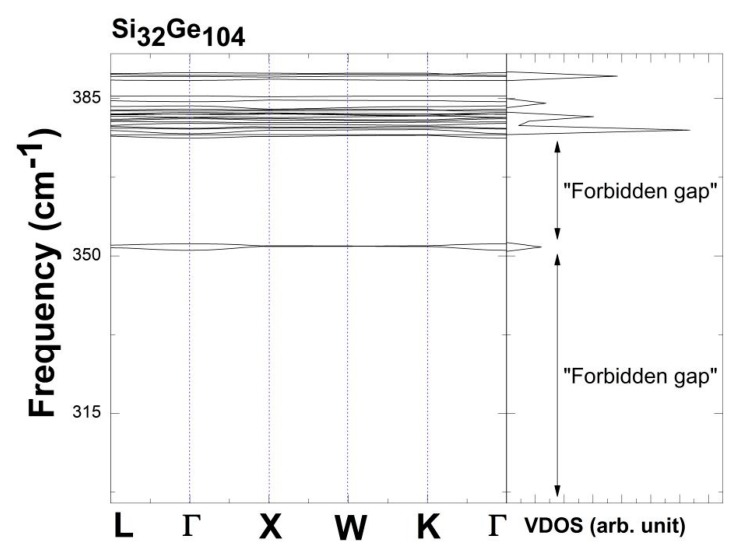
Plot of “forbidden gap” along with the thin peak around 350 cm^−1^ for Si_32_Ge_104_, in terms of dispersion relation and vibrational density of states.

Additionally, one can notify the abrupt change in the dispersion bands from Si_96_Ge_40_ to Si_32_Ge_104_ clathrates of Figure 6. In order to zoom into the smaller phonon energy band widths to identify the “forbidden gap” as well as the thin band level located around 350 cm^−1^, we use Figure 8 to display the vibrational density of states along with the corresponding vibrational spectra confined inside the range of 295 cm^−1^ and 395 cm^−1^. Below the thin peak which is at around 350 cm^−1^, there exists the forbidden gap in the frequency range of 295 cm^−1^ and about 350 cm^−1^. Meanwhile, above 350 cm^−1^, there also demonstrates another “forbidden gap” located within the regime of about 353 cm^−1^ and about 376 cm^−1^.

**Figure 9:**
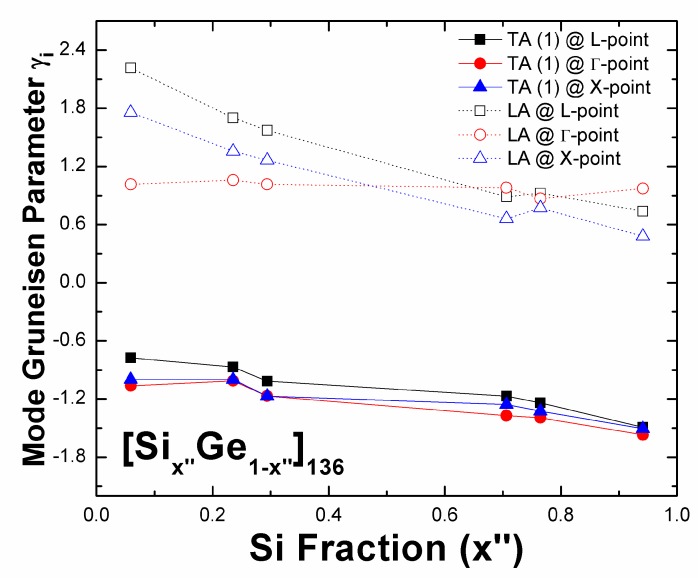
Predicted mode Grüneisen parameters for TA (1) and LA phonons that are confined at the Brillouin zone boundary and center, as functions of the Si content *x”* in [Si_x”_Ge_1-x”_]_136_.

According to the DFT-determined diagram (Figure 9), we see how the number of Ge–Ge bonds that are formed relates to the Si-fraction-dependent mode Grüneisen parameter of TA (1) and LA phonons at various high-symmetry points in [Si_x”_Ge_1-x”_]_136_ (0 < *x” <* 1). Here, *γ*_i_ is computed theoretically via *γ*_i_ = (−*V*/*ω*_i_)(Δ*ω*_i_/Δ*V*) using the finite different method. Despite the almost constant calculated value of *γ*_i_ of an LA phonon that is located near the gamma point, the remaining mode Grüneisen parameters of the same phonon confined to the BZ boundary (L and X points) are positive in sign and exhibit approximately decreasing trends as the number of Ge–Ge bonds decreases from 36 to 8. In addition, the negative value of *γ*_i_ for an acoustic phonon at the zone center or boundary also approximately decreases with increasing Si fraction. The determined ratio of *γ*_TA(1)_(*L*) representing *γ*_i_ of a TA (1) phonon at L-point for Si_32_Ge_104_ to *γ*_TA(1)_(*L*) for Si_104_Ge_32_ is approximately 0.72; hence, the lattice framework exhibits a weak vibrational response upon geometry dilation when the Ge fraction dominates.

**Figure 10:**
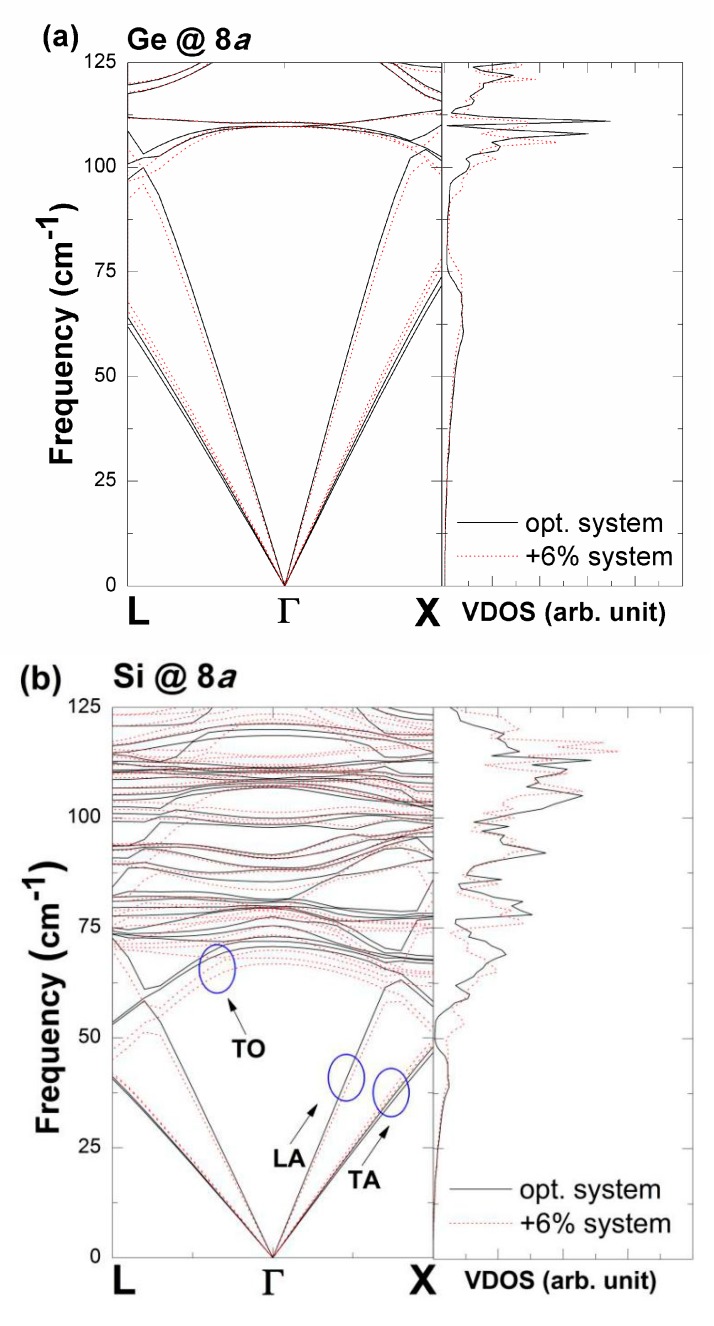
Low-frequency dispersion relation curves of (**a**) Si_128_Ge_8_ (Ge @ 8*a*) and (**b**) Si_8_Ge_128_ (Si @ 8*a*) along the L-Γ-X line, which correspond to the original geometry (solid line) and the dilated configuration (dotted line). LDA-calculated results on the vibrational density of states are also shown. The circled areas correspond to the longitudinal acoustic phonon branch and the transverse acoustic phonon branches along with transverse optical phonon branches with double degeneracy.

The results of the following first-principles calculations (Figure 10) demonstrate the low-energy (0–125 cm^−1^) band structures of the phonon dispersions along the L-Γ-X line for Si_128_Ge_8_ and Si_8_Ge_128_, respectively. To illustrate the dilation geometry effect on the lattice framework anharmonicity, for our plotted phonon spectrum (dashed line), we consider expanded unit cell that is +6% larger than the material’s optimized structure (see “opt. system” in Figure 10a) in Si_128_Ge_8_ to facilitate comparison. Similarly, in Si_8_Ge_128_, the expanded unit cell remains 6% larger than the material’s “opt. system” in Figure 10b. We allow the expanded volume for each material to be +6% times larger than their optimized geometry. This is due to the reason that, the extremely low resolution corresponding to the variation on the wave-vector-dependent phonon mode in the low-frequency *ω*_i_(***q***) regime (such as 0–100 cm^−1^) causes the dispersion relation spectrum difficult to identify, if we use the fractional change in volume that is less than +4%. The red shift of the peak of the vibrational density of states (VDOS) at approximately 68 cm^-1^ in the “opt. system” of Si_8_Ge_128_ is attributable to suppression of its lowest-optic phonon modes (TO branches). A similar red shift of VDOS in optimized Si_128_Ge_8_ is observed for optic phonons, which is in the range of 100 cm^−1^ and 110 cm^−1^.

Thus, the apparent reduction of the mode frequency values for the degenerate TO band in the “+6% system" (Figure 10b), in which the wave-vector spans over the Brillouin zone, results in the existence of positive mode Grüneisen parameters. On the other hand, the phonon frequency for TA branch is elevated in both materials for enlarged geometry, relative to its counterpart (“opt. system” in Si_128_Ge_8_ and “opt. system” in Si_8_Ge_128_). Hence, the value of *γ*_i_(***q***) is negative, which is anticipated to contribute efficiently and dominantly to inducing the low-temperature negative thermal expansion (NTE) phenomenon to occur. 

The exact mode Grüneisen parameters of the specified phonon that are obtained via LDA are listed in Table 2. The measured or theoretically estimated values are obtained at high-symmetry points Γ and L of BZ in the direction given by [111]. It is noted that Wei et al. has reported some predictions [46] on *γ*_i_ of *d*-Si before. All transverse acoustic phonons considered here have *γ*_i_ values that are below zero. The calculated values of *γ*_i_ at the L point for Si_128_Ge_8_ are similar to the experimentally determined values of *γ*_i_ for diamond-phase silicon (see Ref. [35]). The mode Grüneisen parameter of the LA phonon at the Γ point lies between 0.90 and 1.03 for a series of Si_136-x_Ge_x_, thereby resulting in fair comparison with the value of 1.18 that was determined for Na_1_Si_136_ via Raman-scattering experiments. These calculated results also correlate to the *γ*_i_ value of 1.1 that was obtained experimentally for diamond-phase silicon.

In addition to anharmonicity exploration on the low-lying acoustic modes of phonons, our computations demonstrate that the *γ*_i_ values for most of the optical phonon modes are positive. Guided by the quasi-harmonic approximation method, our theoretically derived macroscopic Grüneisen parameter, namely, *γ*(*T*), is the weighted average of mode Grüneisen parameter *γ*_i_, which is expressed as *γ*(*T*) = ∑_i_ *γ*_i_*C*_V,i_/∑_i_ *C*_V,i_ [47,48] where *C*_V,i_ is the partial vibrational mode contribution to the heat capacity. In other words, *γ*(*T*) is related to the anharmonicity of the lattice vibrations and describes how the vibrational frequencies (phonons) change as the volume is varied through *γ*_i_.

**Figure 11:**
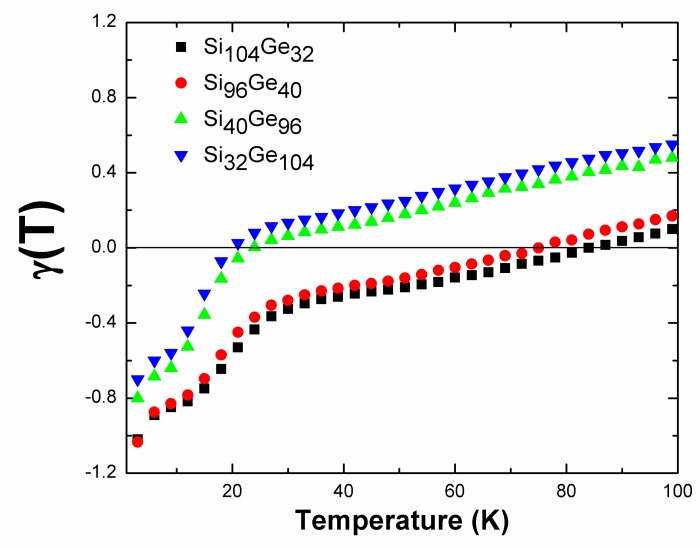
Density functional theory (DFT)-predicted macroscopic Grüneisen parameters of Si_104_Ge_32_, Si_96_Ge_40_, Si_40_Ge_96_ and Si_32_Ge_104_.

In addition, *γ*(*T*) also serves as an indirect tool for surveying anomalous thermal expansion because *γ*(*T*) = *α*_v_(*T*)*K*_T_/*C*_V_*ρ* [49,50] where *α*_v_(*T*) denotes the volumetric thermal expansion coefficient. The sign of *α*_v_(*T*) depends directly on whether *γ*(*T*) is negative or positive since the bulk modulus at the specified temperature *K*_T_ and heat capacity *C*_V,_ along with material’s density *ρ*, always remains positive. The results of our first-principles calculation of the overall Grüneisen parameter for Si_136-x_Ge_x_ (*x* = 32, 40, 96, and 104) is shown in Figure 11, where the axis of abscissa gives rise to a temperature that is limited from 3 K to 99 K. The values of the Grüneisen parameter *γ*(*T*) for Si_104_Ge_32_ and Si_96_Ge_40_ have similar temperature profiles and are always negative from 3 K to approximately 80 K under the scenario of null formation of Ge–Ge bonding. These results on predicting NTE effect can be compared to the reported work of Tang et al., who experimentally and theoretically investigated the thermal properties of Si_136_ and pointed out an NTE region exists between in the 10–140 K temperature range [24]. However, increased numbers of Ge–Ge bonds in Si_40_Ge_96_ and Si_32_Ge_104_ may weaken the NTE effect substantially: the predicted Grüneisen parameters for Si_40_Ge_96_ and Si_32_Ge_104_ remain negative from 3 K to the reduced upper temperature limit, which is approximately 20 K. Further exploration of how the bonding geometry of the Ge–Ge covalent bond (including the bond angle and bond-bond length) impacts the NTE behavior in Si_136-x_Ge_x_ is beyond the scope of this study.

**Figure 12:**
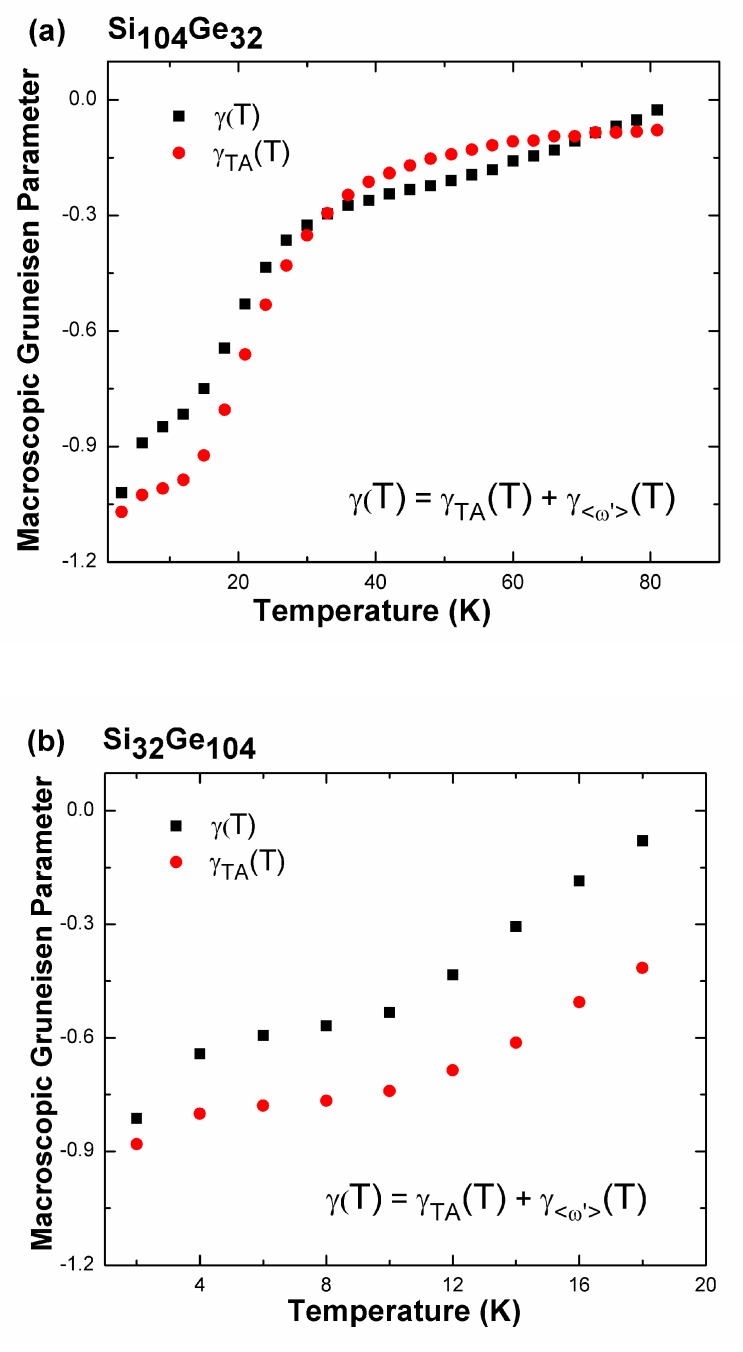
Predicted weighted Grüneisen parameters for transverse acoustic phonons (*γ*_TA_(*T*)) and the contribution of all phonon modes (*γ*(*T*)) as functions of temperature for (**a**) Si_104_Ge_32_ and (**b**) Si_32_Ge_104_.

We decouple the effect of the lowest-lying phonon branches, which contribute to the production of negative mode-dependent Grüneisen parameters, from the contribution of all other phonon modes along all possible high-symmetry directions (see Figure 12a,b). The two lowest phonon bands (transverse acoustic branches), rather than the remaining 100 branches, which are confined to a unit cell of the clathrate system, are anticipated to play a substantial role in producing the NTE phenomenon. Hence, the macroscopic *γ*(*T*) can be calculated primarily from the TA mode contribution via *γ*_TA_(*T*) = *γ*(*T*) − *γ*_<ω’>_(*T*), where *γ*_<ω’>_(*T*), which is relatively small, describes the weighted average of the overall Grüneisen parameter over all optical branches, plus the LA phonon mode contribution. In Figure 12, *γ*_TA_(*T*) dominates *γ*(*T*). It is noted that, the sign of the difference between parameter *γ*_TA_(*T*) and *γ*(*T*) can indicate within what temperature regime, the transverse acoustic phonons may play a much greater role in contributing to the induction of negative thermal expansion behavior than other phonons. As shown in the Figure 12, when the temperature is increasing towards about 80 K in Si_104_Ge_32_ (or 20 K in Si_32_Ge_104_), the temperature-dependent macroscopic Grüneisen parameter approaches almost zero, leading to the vanishing behavior of NTE. Thus, existence of the positive difference (*γ*(*T*) − *γ*_TA_(*T*) > 0) in Figure 12 indicates that, vibration of TA phonons occurring in the temperature range of 0 and 30 K in Si_104_Ge_32_ (or in the range of 0 and 20 K in Si_32_Ge_104_) can contribute more effectively to the induction of NTE than the rest of phonons.

## 4. Conclusions

We have employed the *ab initio* DFT method to conduct systematic investigations on the electronic, vibrational and thermodynamic properties of the Si_136-x_Ge_x_ clathrates. Most of the DFT results relate to vibrational features. We found that low-frequency transverse acoustic phonons, which have an unusual anharmonic vibration response (negative *γ*_i_) to slight structural expansion, are primarily responsible for the occurrence of the NTE phenomenon. In addition, the reduction of the maximum optic band spectrum and the suppression of the acoustic phonon band width are accompanied by an increase in the number of Ge–Ge bonds that are formed, from 0 (or 8) to 36. Moreover, the number of Ge–Ge bonds is expected to affect the upper limit of the temperature range beyond which NTE vanishes, thereby making it possible to have a strongly weakened NTE effect when *x* changes from 32 (or 40) to 96 (or 104) in Si_136-x_Ge_x_. Our structural investigation of Si_136-x_Ge_x_ (0 ≤ *x* ≤ 128) serves as the fundamental step for initiating our entire first-principles work, since all vibrational and thermodynamic properties are extracted, in addition to the optimized geometry of each alloy. Our LDA-determined lattice parameter agrees well with XRD data: both show almost monotonically increasing behavior as the Ge composition, namely, *x*, increases. Regarding the electronic properties, the previous DFT results, which were obtained using the CASTEP code, reveal an optical band gap of Si_128_Ge_8_ of 1.23 eV, which agrees extremely well with the result of our calculation via VASP (~1.27 eV). The tunable band gap modulated by Ge content in Si_136-x_Ge_x_ has attracted attention for photovoltaic (PV) applications, because alloyed SiGe semiconductors demonstrating “nearly-direct” or direct wide band gap may be a very suitable and practical choice for optoelectronics applications [27,37] due to their reduced weight and cost. 

## Figures and Tables

**Table 1:** VASP-predicted vibrational mode frequency of lowest-lying acoustic phonon branch at L, X, W, K high symmetry point for Si_136-x_Ge_x_ (*x* = 8, 40, 104). Numerical values are given in the unit of cm^−1^.

Material	L	X	W	K
Si_128_Ge_8_	61.89	71.81	77.93	73.72
Si_96_Ge_40_	52.41	60.11	65.14	61.86
Si_32_Ge_104_	42.74	49.84	53.90	50.84

**Table 2:** Comparison of mode Grüneisen parameters between experimentally studied Na_1_Si_136_ [35] clathrate along with diamond-phase silicon [35] and theoretically explored Si_136-x_Ge_x_ (8 ≤ *x* ≤ 128), along the Γ-L line ([111]) direction.

Material	Mode	L	Γ
Si_8_Ge_128_	TA (1)	−0.76	−1.04
	LA	2.18	0.98
Si_32_Ge_104_	TA (1)	−0.85	−1.01
	LA	1.68	1.03
Si_96_Ge_40_	TA (1)	−1.16	1.32
	LA	0.87	0.96
Si_104_Ge_32_	TA (1)	−1.20	−1.35
	LA	0.90	0.90
Si_128_Ge_8_	TA (1)	−1.43	−1.51
	LA	0.71	0.93
*d*-Si (Exp.)	TA (1)	−1.30	0.05
	LA	0.90	1.10
Na_1_Si_136_	TA (1)	-----	1.18
